# Prevalence of Hepatitis C virus Genotype 3a in patients with Hodgkin and Non-Hodgkin Lymphoma

**Published:** 2016-12

**Authors:** Hashem Radmehr, Manoochehr Makvandi, Alireza Samarbafzadeh, Ali Teimoori, Niloofar Neisi, Mojtaba Rasti, Sara Abasifar, Hasan Soltani, Samaneh Abbasi, Hadis Kiani, Hamide Mehravaran, Azarakhsh Azaran, Toran Shahani

**Affiliations:** 1 Health Research Institute, Infectious and Tropical Diseases Research Center, Ahvaz Jundishapur University of Medical Sciences, Ahvaz, Iran; 2 Virology Department, School of Medicine, Ahvaz Jundishapur University of Medical Sciences, Ahvaz, Iran; 3 Virology Department, School of Medicine, Kerman University of Medical Sciences, Kerman, Iran

**Keywords:** Nested RT-PCR, Hepatitis C virus, Hodgkin lymphoma, Non-Hodgkin lymphoma, Genotype

## Abstract

**Background and Objectives:**

Hepatitis C virus (HCV) is a major public health problem worldwide. Replication and persistence of HCV genome have been described in the liver tissue as well as B cells lymphocyte. Several investigations have reported that long-term persistence of HCV in B cells may result in Hodgkin and Non-Hodgkin lymphoma. This study was aimed to determine frequency of HCV RNA in histological tissues obtained from patients suffered from Hodgkin and Non-Hodgkin lymphoma.

**Materials and Methods:**

52 formalin-fixed paraffin-embedded tissue blocks including 23 (44.3%) Hodgkin and 29 (55.7%) Non-Hodgkin samples were collected and five micrometer sections were prepared. RNA was extracted and cDNA was synthesized. Two consecutive Nested RT-PCR assays were carried out for detection of HCV 5′ UTR and core gene. RT-PCR products were sequenced and aligned to construct HCV phylogenic tree to evaluate the homology of sequences in comparison to the reference sequences retrieved from Genbank.

**Results:**

Overall, 6 Non-Hodgkin (20.6%) and 3 Hodgkin lymphoma (13.04%) samples showed positive PCR results for both 5′ UTR and HCV core RNA via nested PCR (*P*<0.469). Sequencing results revealed that all detected HCV RNA samples belonged to the genotype 3a.

**Conclusion:**

Despite low prevalence of HCV infection in Iran, high frequency of HCV RNA genotypes 3a (17.3%) has been found in patients with Hodgkin and Non-Hodgkin lymphoma. To improve treatment regimens, screening of HCV RNA in patients suffered from Hodgkin or Non-Hodgkin lymphoma is recommended which can be done through highly sensitive molecular means before and after immunosuppression status.

## INTRODUCTION

Hepatitis C virus (HCV) infection is a major health problem infecting more than 170–200 million people worldwide (1). HCV is hepatotropic and lymphotropic and a causative agent for acute, chronic hepatitis. Persistent chronic HCV may result in cirrhosis and hepatocellular carcinoma (2). HCV belongs to the family of flaviviridae and has a single stranded RNA of 9.7 kb comprises three structural proteins (core, E1 and E2) and seven non-structural proteins (p7, NS2, NS3, NS4A, NS4B, NS5A and NS5B) (3). HCV has been classified into 7 genotypes including over 70 subtypes (4). The rate of HCV infection is varied from 1.5–3.5% in Eastern and Western Europe and more than 3.5% in the Middle East (5). Most patients (80–85%) who become acutely infected cannot clear the infection and will progress to chronic infection. The prevalence of HCV in Iranian general population has been reported less than 1% (6). Different mechanisms have been described the implication of HCV in development of malignant tumors. The expression of HCV core protein (C) and non-structural protein 3 (NS3) will enhance the generation of nitric oxide synthase (NOS) and reactive oxygen species (ROS) and may lead to mutations and abnormalities in DNA repair system and ultimately in cellular transformation (7, 8). HCV NS3 can also bind to tumor suppressor p53 molecule and affect DNA repair process which finally leads to cellular transformation (9). HCV infected marginal B cells lead to chronic stimulation which is thought to result in accumulation of genetic lesions promoting eventually transforming in diffuse large B-cell lymphoma (DLBCL) (10).

Hodgkin’s disease or Hodgkin lymphoma (HL) is characterized by multinuclear Reed-Sternberg cells (RS cells) (11). It has been reported that the role of Epstein-Barr virus (EBV) may increase risk of HL even though the exact mechanism remains unknown (12). Non-Hodgkin’s lymphoma (NHL) is a lymphoproliferative disorder which comprises different hematologic neoplasms that originate from T and B cells in the lymphatic system (13). The etiology of NHL is unknown but may be attributed to several risk factors such as age, sex, genetic and environmental factors (14).

The association of chronic hepatitis C virus (HCV) infection with Hodgkin and non-Hodgkin lymphoma has been reported (15–18). Limited data have been published on the association of HCV infection and Hodgkin and Non-Hodgkin lymphoma in Iran. Thus, this study was conducted to determine the prevalence of HCV RNA in patients with Hodgkin and Non Hodgkin lymphoma in Ahvaz, the capital city of Khuzestan province, located in the south west region of Iran with about 1.5 million population.

## MATERIALS AND METHODS

### Patients

This retrospective study was carried out on 52 paraffin-embedded tissue blocks including 23/52 Hodgkin (44.23%) and 29/52 Non Hodgkin (55.77%) lymphomas. Tissue blocks were collected from archive of Pathology Departments of Shafa and Imam Khomeini Hospitals of Ahvaz during 2001 to 2011. The diagnosis of Hodgkin and Non-Hodgkin lymphoma was done by a pathologist. Clinical data in Table 1 revealed that none of the patients had been tested for HCV RNA, HBV markers, anti-HCV, anti-HIV-1, 2 and anti-HTLV-1 antibodies.

**Table 1.** Profile of patients included in this study

**Category**	**Male %**	**Female %**	**Total %**
Hodgkin Lymphoma: Number 23			
Mixed cellularity (MC)	4(17.39%)	8(34.78%)	12(52.17%)
Lymphocyte Predominant (LP)	3(13.04%)	1(4.34%)	4(17.39%)
Nodular Scleorosis (NS)	3(13.04%)	4(17.39%)	7(30.43%)
Non-Hodgkin Lymphoma: Number 29			
Diffuse Large cell type	4(13.79%)	10(34.48%)	14(48.28%)
Burkitt Lymphoma	2(6.89%)	2(6.89%)	4(13.79%)
Small lymphocytic lymphoma	0	1(3.44%)	1(3.44%)
High grade Immunoblastic lymphoma	1(3.44%)	0	1(3.44%)
Mixed Small and Large	2(6.89%)	0	2(6.89%)
Malignant T Cell	2(6.89%)	2(6.89%)	4(13.79%)
Malignant B Cell	1(3.44%)	1(3.44%)	2(6.89%)
Diffuse Mixed Large Cell	1(3.44%)	0	1(3.44%)
Billirubin (mg/dl)		0.5–1±0.25	
AST (U/L)		24.17±7.3	
ALT (U/L)		41.02±9.3	
HBsAg		Negative	
HCV Ab		Not done	

### Deparaffinization of tissue samples

Initially, sections of 5 μm thickness were prepared from each tissue block followed by deparaffinization. In brief, 800 μl xylene and 400 μl ethanol was added to each sample and centrifuged at 13,000 rpm for 2 min. The supernatant was discarded and 1 ml absolute ethanol was added to the pellet. Tubes were incubated at 55°C for 10 min. 100 μl lysate buffer, containing 16 μl 10% SDS and 40 μl proteinase K, was added to each tube and kept at 55°C overnight (16 hours). The tubes were centrifuged at 13,000 rpm for 2 min and the supernatants were collected and used for RNA extraction.

### RNA extraction and cDNA preparation

Total RNA was extracted from each sample using High pure RNA Paraffin kit (Roche, Germany) according to the manufacturer’s instructions. 10 μl sample was used for cDNA synthesis using available commercial kits (Thermo Fisher Scientific, Latvia) according to the manufacturer’s instructions.

### Nested PCR for 5′ UTR

**Fig. 1.**
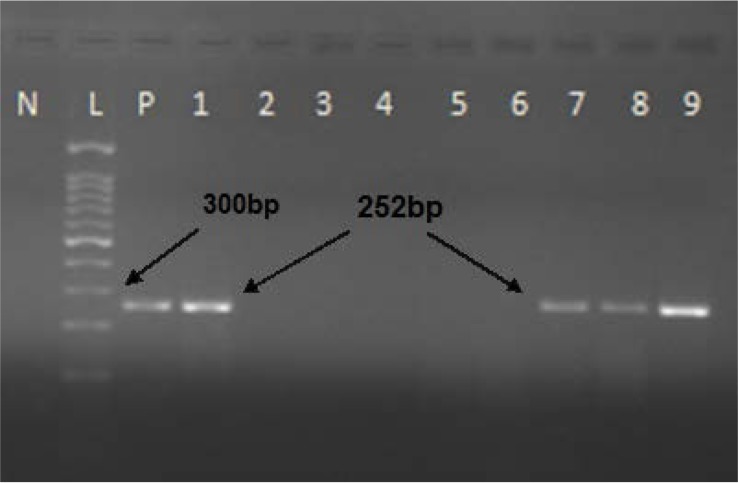
HCV 5′UTR PCR. N: Negative control; L: 100 bp DNA ladder; P: Positive control; #1 to 9: Unknown samples

Detection of HCV RNA was carried out using cDNA samples via a nested RT-PCR. The following primers from 5′ untranslated region (UTR) of HCV genome were used; for the first PCR: BKP 7 (outer sense primer) (nucleotides 38–62; 5′-cactcccctgtgaggaactactgtc-3′), BKP 8 (outer anti-sense primer) (nucleotides 319 to 343) (5′-atggtgcacggtctacgagacctcc-3′). 25 microliter reaction mixture was prepared from 2.5 μl 10X PCR buffer (Roche), 0.5 μl dNTP mix (0.2 mM), 1 μl each primer (BKP-7 and BKP-8) (0.5 μM), 0.15 μl Taq DNA polymerase (1 Unit), 7 μl cDNA template and water. Positive and negative controls were also included in each PCR. PCR was programmed by initial denaturation at 95°C (5 min) followed by 35 cycles of 94°C (1 min), 60°C (1 min), 72°C (1 min) and final extension at 72°C (10 min). The second round PCR was performed similar to the first PCR using the same PCR reaction mixture with inner set of primers (BKP-9 and BKP-10) (BKP 9; nucleotides 63 to 87: 5′-ttcacgcagaaagcgtctagccatg-3′; BKP 10; nucleotides 292 to 314: 5′-gcgcactcgcaagcaccctatcagg-3′). PCR conditions were the same as the first round for 30 cycles. The first PCR product was 306 bp in length whereas the second product was 252 bp (19) (Fig. 1).

### Nested PCR for core of HCV

**Fig. 2.**
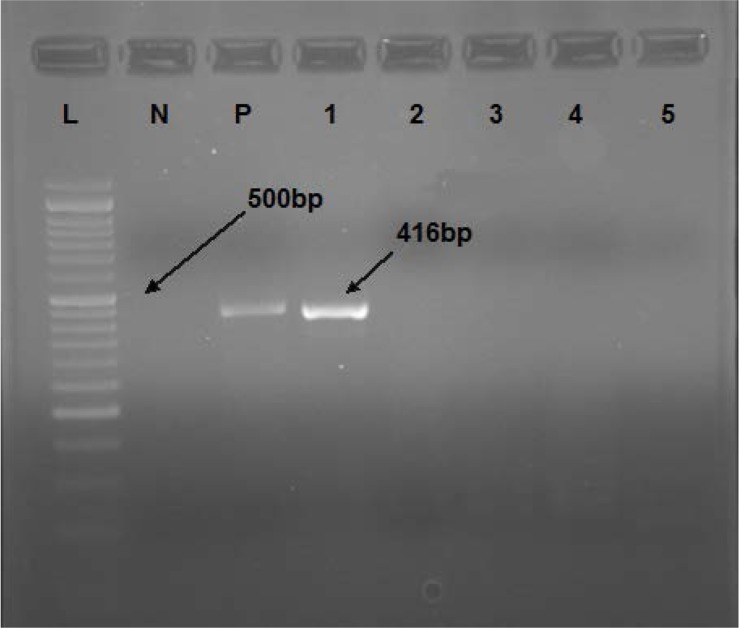
HCV core PCR. L: 50 bp DNA ladder; N: Negative control; P: Positive control; #1–5: Unknown samples

Second nested PCR was fulfilled for detection of core gene of HCV in tissue samples with the following primers: SC2 (F primer; 5′-gggaggtctcgtagaccgtgcaccatg-3′), AC2 (R primer; 5′-gagmggkatrtaccccatgagrtcggc-3′), S7 (F primer; 5′-agaccgtgcaccatgagcac-3′) and 584 (R primer; 5′-cccatgaggtcggcraarc-3′). PCR reaction mix was prepared from 2.5 μl 10X PCR Buffer (Roche), 0.5 μl dNTP mix (0.2 mM), 1 μl forward and reverse primers (0.5 μM), 0.15 μl Taq DNA pol (1 Unit), 7 μl cDNA template and water. PCR condition was programmed as follows: initial denaturation at 95°C (5 min) and 35 cycles of 95°C (1 min), 55°C (1 min), 72°C (1 min) and final extension at 72°C (10 min). For the second PCR, the reaction mixture and PCR temperatures were programmed similar to the first PCR. 416 bp PCR products on 2% agarose gel indicated the positive reactions (Fig. 2) (20).

### Sequencing

To determine the genotypes of HCV samples, 9 positive samples of 5′ UTR and core regions were sequenced. The sequences were blasted using available databases. A phylogenic tree was constructed with Neighbor joining method using the partial nucleotide sequences of 5′ UTR region of HCV positive samples. Reference sequences were retrieved from GenBank using their accession numbers.

### Statistics

Data analysis was done by SPSS (v. 17.0) using mean, standard deviation (SD), and Chi-square test. P values < 0.05 were considered as significant.

## RESULTS

**Fig. 3.**
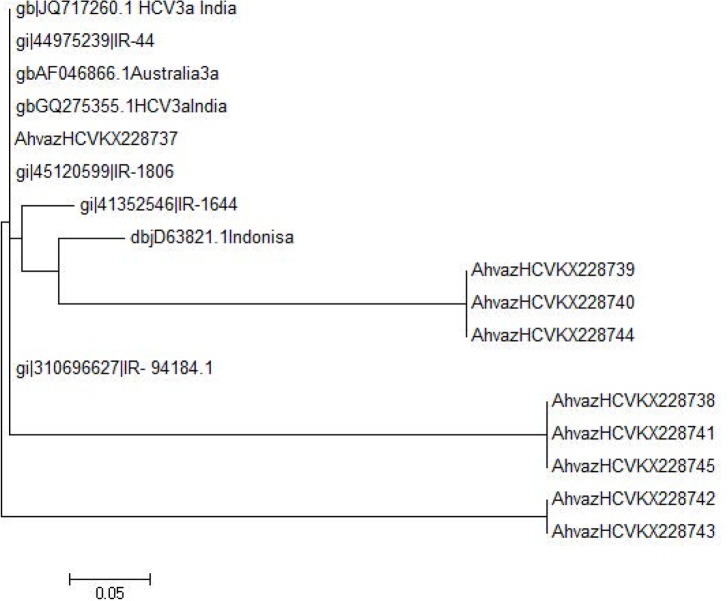
A phylogenic tree was constructed using the partial nucleotide sequences of 5′ UTR regions of the isolated HCV genotype 3a. Reference sequences were retrieved from Gen-Bank with their accession numbers. Numbers in branch are reproducibility after 1000 bootstraps. Scale bar = 5%

The age of the patients ranged from 5.48 to 34 years with the mean age of 19.74 ± 14.2 years. In overall 52 samples, 9 samples (17.3%) represented positive 5′ UTR and HCV core (P=0.469) including 6 Non-Hodgkin (6 out of 29; 20.68%; 4 males and 2 females) and 3 Hodgkin (3 out of 23; 13.04%; 1 male and 2 females) lymphomas (P=0.469). Sequence analysis revealed that all positive HCV RNA samples belonged to genotype 3a (Fig. 3). Table 2 also represented the distribution of HCV infection in subtypes of Hodgkin and Non-Hodgkin lymphomas.

**Table 2.** Distribution of HCV RNA in Hodgkin and Non-Hogkin Lymphoma subtypes

**Lymphoma**	**Subtypes**	**HCV RNA positivity (%)**
Hodgkin (N=23)	Mixed Cellularity (MC) (N=12)	2 (8.69)
	Lymphocyte Predominant(LP) (N=4)	1 (4.34)
Non-Hodgkin (N=29)	Diffuse Large Cell (N=14)	2 (6.89)
	Malignant T cell (N=4)	1 (3.44)
	Malignant B cell (N=2)	1 (6.89)
	Burkitt (N=4)	2 (6.89)

## DISCUSSION

In our study 6 Non-Hodgkin (20.6%) and 3 Hodgkin (13.04%) lymphoma samples showed positive PCR results for 5′ UTR and core regions of HCV. The prevalence of HCV infection in patients with Hodgkin lymphoma is controversial. In 2003, Keresztes et al. have detected HCV RNA in 10 out of 111 Hodgkin lymphoma patients (9%) in Hungary (21). Dal Maso et al. have also found HCV RNA in 1.6% of Hodgkin patients (22). In 2015, Tehseen Iqbal et al. have observed HCV RNA in 44% of Hodgkin lymphoma patients in US (23). We have detected HCV RNA in 13.04% of Hodgkin lymphoma patients which indicated that our finding situated between the above findings. HCV RNA has also been detected in patients with Non-Hodgkin lymphoma. The rate of HCV RNA detection among patients with Non-Hodgkin lymphoma is varied from 2.4% in Canada to 42.7% in Egypt. In Egypt, Cowgill et al. have reported a high prevalence of HCV RNA (42.7%) in Hodgkin lymphoma patients (24) while Spinelli et al. have detected 2.4% HCV RNA positive cases among Canadian Non-Hodgkin lymphoma patients (25). John et al. in 2008 reported 17.5% HCV RNA positive Non-Hodgkin lymphoma cases in Italy (26). We have detected 20.68% HCV RNA in Non-Hodgkin lymphoma patients. The overall HCV RNA positivity in the present study was 3 out of 23 Hodgkin lymphoma (13.04%) and 6 out of 29 (20.6%) Non-Hodgkin Lymphoma (P= 0.469). The HCV genotypes 1a, 3ad, 1b, 2 and 4 have been reported in Iran (27, 28).

We have detected HCV genotype 3a in 2/23 (8.69%) patients with Hodgkin Mixed Cellularity (MC) subtype, and 1/23(4.34%) in patient with Lymphocyte Predominant (LP) whereas Katalin et al. in Hungary detected HCV RNA in 10/63(15.87%) patients suffered from Hodgkin mixed cellularity subtype (MC), 7/38(18.42%) nodular scleorosis (NS) and 2/8(25%) lymphocyte-depleted group (LD) (21).

In the present study, we detected HCV genotype 3a in Non-Hodgkin subtypes including 2(6.89%) in diffuse large cell group, 2(6.89%) in Burkitt Lymphoma, 1(3.44%) in malignant T-cell group and 1(3.44%) in malignant B cell group. Visco et al. have detected HCV RNA in diffuse large B-cell lymphoma (DLBCL) subtype of Non-Hodgkin lymphoma (29). Several studies have been strengthened that antiviral therapy against HCV in patients with low grade B cell lymphoma was very effective (30, 31).

## CONCLUSION

Despite low prevalence of HCV in Iran, high frequency of HCV RNA genotype 3a(17.3%) has been found in patients suffered from Hodgkin and Non-Hodgkin lymphomas. In the light of aforementioned data it is recommended that for improving treatment regimens, screening of HCV RNA should be implemented for Hodgkin and Non-Hodgkin lymphoma patients by highly sensitive molecular means before and after immunosuppression status.
